# Bioinspired and Biomimetic Nanomedicines for Targeted Cancer Therapy

**DOI:** 10.3390/pharmaceutics14051109

**Published:** 2022-05-23

**Authors:** Xiaoqiu Xu, Tong Li, Ke Jin

**Affiliations:** 1 Laboratory of Human Diseases and Immunotherapies, West China Hospital, Sichuan University, Chengdu 610041, China; xqxu2020@wchscu.cn (X.X.); tongli@scu.edu.cn (T.L.); 2 Institute of Immunology and Inflammation, Frontiers Science Center for Disease-Related Molecular Network, West China Hospital, Sichuan University, Chengdu 610041, China; 3 State Key Laboratory of Biotherapy and Cancer Center, West China Hospital, Sichuan University and Collaborative Innovation Center for Biotherapy, Chengdu 610041, China

**Keywords:** targeted drug delivery, biomimetic strategy, bioinspired nanomedicine, biohybrid nanoparticles, cancer treatment

## Abstract

Undesirable side effects and multidrug resistance are the major obstacles in conventional chemotherapy towards cancers. Nanomedicines provide alternative strategies for tumor-targeted therapy due to their inherent properties, such as nanoscale size and tunable surface features. However, the applications of nanomedicines are hampered in vivo due to intrinsic disadvantages, such as poor abilities to cross biological barriers and unexpected off-target effects. Fortunately, biomimetic nanomedicines are emerging as promising therapeutics to maximize anti-tumor efficacy with minimal adverse effects due to their good biocompatibility and high accumulation abilities. These bioengineered agents incorporate both the physicochemical properties of diverse functional materials and the advantages of biological materials to achieve desired purposes, such as prolonged circulation time, specific targeting of tumor cells, and immune modulation. Among biological materials, mammalian cells (such as red blood cells, macrophages, monocytes, and neutrophils) and pathogens (such as viruses, bacteria, and fungi) are the functional components most often used to confer synthetic nanoparticles with the complex functionalities necessary for effective nano-biointeractions. In this review, we focus on recent advances in the development of bioinspired and biomimetic nanomedicines (such as mammalian cell-based drug delivery systems and pathogen-based nanoparticles) for targeted cancer therapy. We also discuss the biological influences and limitations of synthetic materials on the therapeutic effects and targeted efficacies of various nanomedicines.

## 1. Introduction

Tumor progression and recurrence are leading causes of mortality. An estimated 19.3 million new cases and almost 10 million cancer deaths occurred worldwide in 2020 [1]. Currently, the diagnosis and treatment of cancer are hampered by the complexity and heterogeneity of tumor biology and an incomplete understanding of therapeutic interactions within biological systems [2,3,4]. Generally, most conventional chemotherapeutics will cause non-specific systemic biodistributions of drugs which can induce intolerable cytotoxicity to healthy tissues [5]. Hence, cancer therapies are often accompanied by many unwanted side effects associated with free chemotherapeutic drugs, such as myelosuppression, anemia, thrombocytopenia, mucositis, and organ dysfunction [6,7,8]. In addition to their unfavorable biodistribution and poor selectivity, insufficient drug concentrations at the tumor site and multiple drug resistance largely reduce the efficacy of cancer therapy [9]. Therefore, targeted delivery is of utmost importance in order to overcome current limitations in cancer therapy [10,11].

In past decades, many promising materials for biomedical applications have been developed with the progress of nanotechnology, which has caused a boost in the development of various therapies. Among these therapies, nanomedicine has been extensively explored to improve the diagnosis and treatment of cancers due to its exclusive physicochemical properties, such as nanoscale size, large surface area-to-volume ratio, tunable surface features, and ability to encapsulate various drugs and control drug release [2,12]. Compared to conventional chemotherapeutic drugs, nanomedicines play a more prominent role in prolonging circulation half-lives and increasing the stability, bioavailability, and tumor accumulation of drugs [13,14,15]. These tunable physiochemical properties can be employed in targeted strategies to improve the biodistribution and target-site accumulation of free drugs, improving on conventional cancer treatment in terms of specificity [16,17].

Improving tumor targeting efficiency is the critical problem and the major technical difficulty in developing superior tumor-targeting nanodrugs. Passive tumor targeting via the enhanced permeability and retention (EPR) effect has long been considered the most effective mechanism for the accumulation of nanoparticles (NPs) in solid tumors; however, whether it works as a key cornerstone of tumor-targeted drug delivery has long been controversial [11,18,19]. The real efficiency of targeted NPs varies due to the heterogeneity of the EPR effect [19,20]. Compared to the EPR effect, surface functionalization with high-affinity ligands, which has been developed as an active targeting strategy to improve therapeutic efficiency, enables nanomedicines to easily penetrate tumor sites [21,22]. This ligand-mediated method utilizes affinity ligands on the surface of delivery carriers to bind specific molecules or receptors overexpressed in pathological organs, tissues, cells, and even subcellular domains [23,24]. Therefore, these NPs with tailored surfaces have the potential for prolonged circulation time, enhanced immune evasion, and superior targeting capacity [25,26]. However, numerous challenges still lie ahead for clinical translation, though substantial advancements have been achieved in the development of active targeting strategies [2]. For instance, in preclinical studies, drug-loaded nanocarriers can reach tumor sites to achieve maximal therapeutic efficacy with minimal toxic effects, but in clinical practice almost none of the active targeted NPs work due to impenetrable biological barriers, resulting in no approved nanocarrier-based drugs being available for clinical use [2]. This predicament is largely due to the complexity and heterogeneity of tumors, and the artificial properties of synthetic NPs mean that they lack the intrinsic chemotactic features necessary to overcome biological barriers easily [11].

With deeper understanding of biological and physiological systems and further development of nanotechnology, scientists have gained inspiration from natural structures and processes in the living world and can synthesize versatile and effective nanomedicines mimicking biological features to overcome various biological barriers [27,28]. Moreover, some biomimetic features can also be incorporated into synthetic materials to make nanomedicines with controlled size, shape, and surface parameters and to achieve specific therapeutic goals [29,30]. In addition to these characteristics, bioinspired nanomedicines are biocompatible, biodegradable, and have a superior capability to circulate for payload delivery and potential therapeutic improvement. Therefore, bioinspired nanomedicines are providing new strategies to overcome biological barriers and to better solve other shortcomings of current drug delivery systems (DDSs).

**Figure 1:**
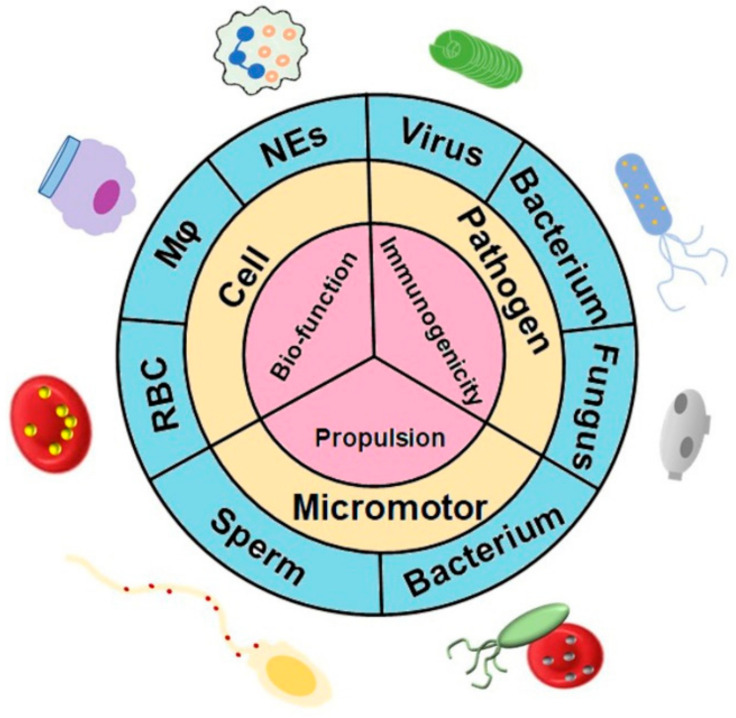
Bioinspired and biomimetic nanomedicines for targeted drug delivery. A variety of natural living systems have been used as sources of carriers for targeted delivery of therapeutic agents. These biohybrid drug delivery systems not only preserve the physicochemical properties of the synthetic materials but also provide unique biofunctionalities supplied by integrated cells. The strategies of targeted delivery systems can be adapted according to the desired applications. Abbreviations: Mφ, macrophage; NEs, neutrophils; RBC, red blood cell.

In this review, we narrow our focus to recent advances in bioinspired nanomedicine for targeted delivery based on various natural and synthetic materials. The applications and limitations of each approach will also be highlighted and discussed (Figure 1).

## 2. Physicochemical Impact on Delivery Efficiency

### 2.1. Size

**Figure 2:**
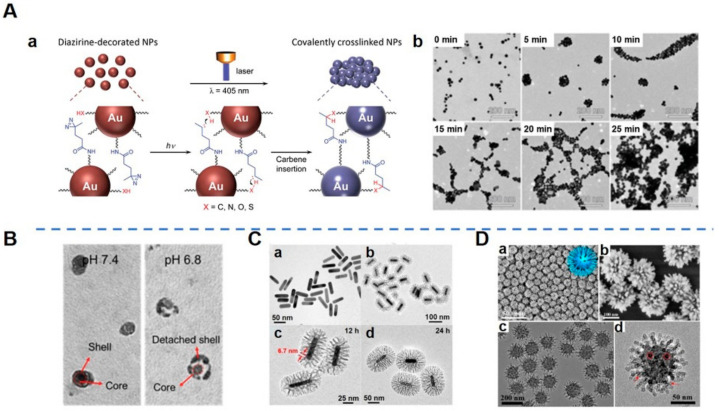
Morphological design of nanoparticles (NPs). (**A**). Light-triggered assembly of gold NPs (AuNPs). (**Aa**) Schematic illustration of a light-triggered assembly of diazirine-decorated AuNPs (dAuNPs). (**Ab**) Transmission electron microscopy (TEM) images of dAuNPs before and after illumination with a 405 nm laser for different periods of time [42]. Aggregation and the agglomeration degree of dAuNPs depended on irradiation time, demonstrating that interparticle cross-linking took place upon laser irradiation. (**B**). TEM images of shell-stacked NPs (SNPs) in PBS at pH 7.4 or 6.8 [45]. SNPs with size and charge dual-transformable ability displayed a clear spherical core–shell structure at pH 7.4, with a size of 145 nm. When SNPs were incubated at pH 6.8, a polyethylene glycol (PEG) corona detached from the core and subsequently the small-sized core with a size of 40 nm was exposed. (**C**). Morphology and structure of gold nanorods (GNRs) and bacteria-like mesoporous silica nanoshell (MSN)-coated GNRs (bGNR@MSN). (**Ca**) TEM image of GNRs. (**Cb**,**Cc**) TEM images of bGNR@MSN coated for 12 h with silica. The red arrows indicate the size (~6.7 nm) of mesopores. (**Cd**) TEM image of bGNR@MSN coated for 24 h with silica [29]. The morphology of the outside mesoporous silica layer resembled bacterial pili, and the thickness of the mesoporous silica layer could be controlled by changing the reaction time. (**D**). Morphology of virus-like mesoporous silica NPs. (**Da**,**Db**) Scanning electron microscopy (SEM) and (**Dc**,**Dd**) TEM images with different magnifications of the virus-like mesoporous silica NPs. The red arrows mark the open tubular structures; the red circles highlight the top view of the open silica nanotubes. The inset of (**Da**) is a structural model for the virus-like mesoporous silica [47]. (Image (**A**) is reproduced with permission from [42] (Copyright © 2016 John Wiley & Sons, Inc.). Image (**B**) is reproduced with permission from [45] (Copyright © 2017 John Wiley & Sons, Inc.). Image (**C**) is reprinted with permission from [29] (Copyright © 2018 Elsevier Ltd.). Image (**D**) is reprinted with permission from [47] (Copyright © 2017 American Chemical Society).)

As one of the most important characteristics of NPs, size greatly influences their biodistribution after systemic administration [31]. There is a close correlation between anti-tumor effect and the size of nanomedicines [32]. Studies have found that NPs of larger size tend to remain in tumor tissues longer than those of smaller size [21]. Conversely, though smaller particles have a better penetration ability, they are eliminated rapidly [33,34,35]. That is, the smaller particles probably have a higher permeability with respect to tumor sites but cannot guarantee enhanced accumulation because they can be easily pumped back into the bloodstream by the high interstitial fluid pressure of the tumor [36,37]. Therefore, researchers have gradually realized that a compromise should be made regarding the “size dilemma”—that there is an equilibrium point between penetration and retention [38]. Smart size-tunable strategies, including stimuli-induced aggregation and shrinkage, provide a potential method for optimizing the size of NPs to enhance their retention and penetration in tumor sites in response to various stimuli in the tumor microenvironment (TME), including enzymes, redox, temperature, light, and pH [35,39,40,41,42,43,44]. Aggregation strategies using small-sized NPs for deep penetration produce large agglomerates for enhanced retention once NPs penetrate the tumor after specific stimulations [38] (Figure 2A), while shrinkage can be used to generate NPs with a smaller size to deeply penetrate and be retained in tumors. Theses NPs exhibit merits in terms of nuclear delivery, drug release, renal clearance, and secondary distribution [45,46]. Hence, there is a question: which strategy is better, given that the two strategies are opposite? To answer this question, sufficient experiments need to be carried out to validate these methods. Alternatively, a flexible strategy may be a better option. For example, Chen et al. developed an excellent NP which could maintain a certain size and negative surface charge for prolonged circulation [45]. After accumulating in the acidic TME, these NPs shrank to a smaller size and switched to a positive surface charge for efficient penetration and retention in the interstitial space throughout the tumor tissue [45] (Figure 2B). The design of such size and charge dual-transformable nanomedicine is an attempt to explore ways of making the most of the physiochemical features of nanomedicines.

### 2.2. Shape

Spheres remain the predominant shapes of particulates because they are easy to manufacture [48]. Non-spherical shapes, including rod-like, disc-like, and other unusual morphologies inspired by natural entities, also exhibit unique properties which are influenced by specific structure–activity relationships between particle shapes and biological processes [49]. It has been demonstrated that ellipsoid particles display lower internalization by macrophages than spheres with prolonged circulation time [50]. Inspired by the intrinsic surface features of the natural biomembrane system, many particles with biomimetic shapes have been reported to exhibit functional merits. For example, bacteria-like mesoporous silica nanoshell-coated gold nanorods present high doxorubicin (DOX) loading efficiency (40.9%, *w*/*w*) due to a large pore volume and surface area [29] (Figure 2C). Another example of a unique virus-like mesoporous silica NP has a spiky, tubular, and rough surface, which is associated with high cellular uptake (the particles invade living cells in large quantities within 5 min) [47] (Figure 2D). Nie et al. designed a spiky nanoinhibitor which can neutralize influenza A virus by matching the surface topology of the viral particle to block its attachment and entry to host cells [51]. The reason why researchers continue to study shape effects is that they want to figure out whether shape effects can offer benefits for specific therapeutic purposes; therefore, specifications of definite mechanisms for nano-biointeractions should be verified by reliable experiments.

### 2.3. Permeability and Retention

With an in-depth understanding of the interaction between nanomedicines and biosystems, pathological features of cancers and TME have been elucidated and exploited for targeted drug delivery in cancer treatment. In particular, the EPR effect of solid tumors has been extensively studied and utilized for passive targeting therapy. The EPR effect is the fundamental pathophysiological phenomenon that occurs in solid tumors and it is universally observed in human cancers [52]. This strategy is based on abnormal vasculatures in which endothelia are fenestrated, with gaps between 100 nm and 780 nm of size, enabling the extravasation and retention of macromolecules and nanocarriers at pathological sites [53]. However, a debate has been provoked in recent years about the real efficiency of the EPR effect due to its large inter- and intraindividual heterogeneity [20]. Wilhelm et al. claimed that a median of 0.7% of the administered dose of the NPs under study was detected in solid tumors in mouse models and that the median delivery efficiency had not improved in the past 10 years [11]. Other experts argued that this negative consequence relied largely on an unconventional parameter being selected for efficiency evaluation without consideration of the active pharmaceutical ingredient [54]. Ding et al. confirmed that 87.8% of the human renal tumors in their study showed a considerable EPR effect, although significant diversity and heterogeneity was exhibited in an ex vivo perfusion model [55]. Another study indicated that NPs were transported into tumors predominantly by active transendothelial mechanisms instead of a passive process because tumor vessels were mostly continuous and interendothelial gaps rarely occurred, even though these gaps had a size of up to 2000 nm, which was sufficient to allow NPs to enter tumors [53,56,57]. Regardless of whether the localization of nanocarriers in tumors is mainly attributed to the EPR effect, passive accumulation, though it definitely occurs after systemic administration, is highly heterogeneous, both inter- and intraindividually [17,55]. Despite this, nanocarriers themselves are frequently utilized to transport therapeutic drugs to the tumor site due to their nanoscale size advantage.

Overall, in spite of the current controversy regarding its heterogeneity and efficacy, the EPR effect is still considered an effective means and a fundamental basis for delivering nanomedicines to tumors. It is critical to further identify the pathophysiological mechanisms and limitations of the EPR effect in order to better utilize this concept in developing tumor-selective nanomedicine delivery strategies and thereby achieve satisfactory outcomes in clinical applications [52,58].

### 2.4. Stimuli-Triggered Drug Release

Stimuli-responsive drug release is a pivotal strategy in current drug delivery research due to the “smart” drug release behaviors of certain types of nanomedicines at predictable locations in response to local (i.e., physiological or pathophysiological) cues, including pH, redox, hypoxia, glucose, and enzymes, or in response to external stimuli, such as light, ultrasound, magnetic fields, electric fields, and temperature [59,60,61,62,63,64]. These so-called “smart” therapeutics mainly depend on hydrolysis-controlled release systems. They can sensitively alter their microstructures to adapt to minor environmental changes to reduce the systemic toxicity and enhance the efficiency of cancer therapy [59]. In addition, dual- or multiresponsive nanomedicines may provide a better option because they possess more comprehensive abilities of site-specific drug release in response to the complex, heterogeneous, and dynamic TME for improved cancer treatment. For example, Huang et al. developed a kind of smart nanoparticle which was dual-triggered by pH and matrix metalloproteinase 2 [65]. Song et al. engineered a unique smart nanoagent based on self-assembled quantum dots–phenolic nanoclusters for improved antitumor chemotherapy through adenosine triphosphate (ATP)-responsive drug release in cancer cells [66]. Although considerable effort has been expended, the development of responsive nanomedicines still has a way to go.

## 3. Mammalian Cell-Based DDSs

The research field of drug delivery optimization has grown tremendously in the past few decades with the development of nano-sized DDSs. The synthetic materials, either organic or inorganic, may have defects in terms of biocompatibility and tumor targeting, such as difficulties in crossing biological barriers and short blood circulation half-lives. Compared to synthetic materials, biomimetic nanomedicines originated from natural cells possess various advantages owing to their high biocompatibilities and versatile functionalities, which can compensate the limitations of synthetic materials to some extent.

### 3.1. Cell Membrane-Camouflaged NPs

Cell membrane-camouflaged nanomedicines not only preserve the physicochemical properties of the synthetic materials, they also inherit biological functions from the original source cells due to integrated membrane compositions with surface molecular diversity [67,68]. The surface properties and complex biofunctions of biomimetic NPs inherited from source cells are difficult to replicate with traditional chemical modifications. Several methods have been developed to construct membrane-cloaked nanomaterials with many types of membranes. Generally, the fabrication process of cell membrane-coated NPs is facile, which includes two main steps: isolating the cell membrane and fusing the membrane with the synthetic core [69]. With regard to erythrocytes and platelets, which are both devoid of nuclei, we can directly extract membrane vesicles by high-speed centrifugation. Regarding other nucleated cells, complex biochemical procedures are often needed to completely remove intracellular contents, including hypotonic lysis followed by ultrasonication, along with discontinuous sucrose density centrifugation, repeated freeze–thaw cycles and so on [70,71]. Afterwards, the synthesized cores can be covered with the membrane using different approaches. Among these strategies, the most frequently used method is physical extrusion, by which the synthesized core and the membrane are co-extruded to pass through polycarbonate porous membranes of the desired size [30]. Although the extrusion process is quite promising, lab productivity performance has been disappointing, resulting in great challenges for large-scale industrial production, while sonication seems to be more effective and practicable [72]. The types of cell sources, fabrication processes, unique properties, and potential applications of currently available cell membrane-camouflaged nanomedicines are summarized in Table 1. Cell membrane-camouflaged NPs have great potential to overcome biological barriers for active targeting. The integrity of the cell membrane coating on NPs is a critical metric by means of which to evaluate the potency of these biomimetic NPs. Liu et al. uncovered that up to 90% of biomimetic NPs were only partially coated [73]. Whether these partially coated NPs have defective biomedical functions remains unclear and needs further experimental validations.

### 3.2. Whole Cell as Drug Carrier

Compared to the cell membrane-coating strategy which has little component loss, the whole living cell presents an intact system for biological applications with no need for further modifications. Therapeutic cargos can be encapsulated into cells by various methods, including electroporation, diffusion, extrusion, and active endocytosis, depending on the properties of payloads and cell types. Many types of cells can be directly used as whole-cell carriers for drug delivery. For example, due to superior circulation lifespan and high biocompatibility, erythrocytes are frequently used to increase the half-life of drugs in the circulation; phagocytes can be used for drug loading because of their endocytosis and exocytosis functions; certain types of cells that have intrinsic tumor-tropic properties can be used for targeted drug delivery to target certain types of tumors [85,86,87]. In addition, the outer surface of cells offers robust functional groups of molecules, such as proteins, polysaccharides, and lipids, providing diverse synthetic materials for durable adhesion by covalent and non-covalent bindings [88,89].

#### 3.2.1. Red Blood Cells

Red blood cells (RBCs) have a biconcave disc shape, maximized surface area-to-volume ratio, and a deformable soft cytoskeleton, enabling them to be resistant to lysis and fracture and to squeeze through constricted spaces within the vasculature [90,91]. In addition, some RBC membrane glycoproteins can protect RBCs from damage and elimination from the bloodstream by the immune system, allowing human RBCs to circulate in vivo for a long period of time (~120 days) [91]. RBCs also have many other advantages, such as considerable total amount in circulation, easy collection, and high drug-loading capacity [92]. Owing to these favorable features, RBCs are widely used to deliver drugs for long-term release in blood circulation, with high drug-loading, good biocompatibility, and limited immunogenicity [90,93].

RBC-derived pharmaceutical nanocarriers have gained remarkable interest in past decades for use in the development of targeted cancer treatment. This is partially due to the potential for improved passive tumor-targeting via the enhanced EPR effect. Several chemical or physical methods have been developed to load drugs in RBCs or attach them on the outer surfaces of RBCs. For example, the osmotic lysis-based method is a common strategy to load therapeutic agents into RBCs. It is based on the basic principle that RBCs swell in hypotonic drug solutions, this being followed by pore formation on the cell membranes, which allows the diffusion of dissolved substances into cells driven by a concentration gradient [94,95]. The pores will be partially resealed in the hypertonic solutions and the substances will be retained in the RBCs at appropriate concentrations [94]. L-asparaginase (ASNase)-loaded RBCs are the typical example of entities prepared by this method. ASNase-loaded RBCs can specifically catalyze the substrate asparagine (ASN) to produce free L-aspartic acid and circulate for a longer time as enzyme bioreactors [96,97]. What is more, research on ASNase-loaded RBCs has entered the clinical stage for treatment of acute lymphoblastic leukemia, pancreatic cancer, acute myeloid leukemia, and triple-negative breast cancer [96]. However, despite the great clinical progress of ASNase-loaded RBCs, the osmotic lysis-based method still has several limitations. Although the pores in cell membranes can be partly restored, the damage is irreversible, resulting in deterioration of the structural integrity of RBCs and inevitable drug diffusion [97]. To overcome this obstacle and optimize the methodology, researchers have applied non-invasive cell-penetrating peptide-mediated cell internalization to encapsulate ASNase into RBCs, which could maintain the structural integrity of RBC membranes [97]. Other drug-loading methods, such as electroporation, extrusion, and mechanical force (fluidic shear stress), also induce irreversible membrane integrity disruption in RBCs and thus may impair their survival, circulation, and therapeutic functions [94,98]. However, the concept of applying cell-penetrating peptides has stimulated the exploration of other strategies which might improve the existing methods of drug encapsulation.

**Figure 3:**
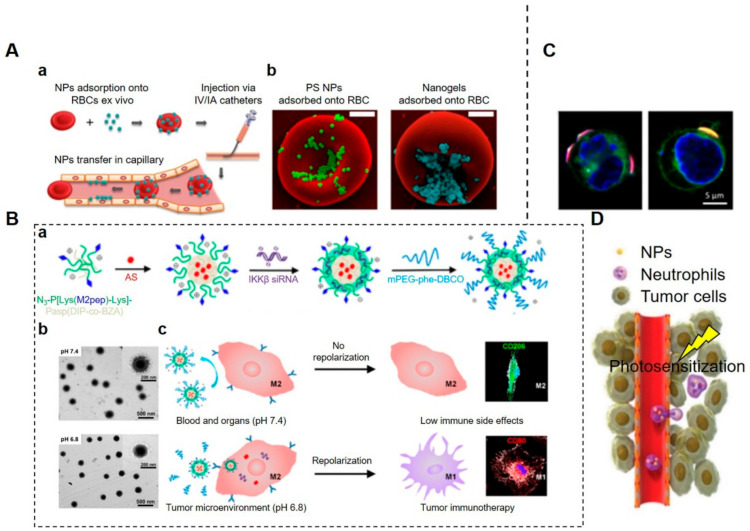
Mammalian cells for targeted drug delivery. (**A**). RBC hitchhiking (RH) drug delivery system. (**Aa**) Procedural steps of RH. NPs were first adsorbed onto the RBCs ex vivo. The RBC–NP complexes were then injected intravenously (IV) or intra-arterially (IA). Then, RH transferred NPs to the capillaries of the first downstream organ. (**Ab**) SEM images of polystyrene NPs (PS-NPs) and nanogels attached to the surface of murine RBCs [100]. NPs were mixed with RBCs in vitro. Scale bars = 1 μm. (**B**). M2-like tumor-associated macrophage (TAM)-targeted NPs. (**Ba**) Composition and structure of a self-assembled micelleplex. The amphiphilic diblock copolymers self-assembled into M2-targeting micelles with therapeutic agents. (**Bb**) TEM images of a micelleplex at pH 7.4 and pH 6.8. At pH 6.8, the size of the micelleplex decreased due to the removal of the sheddable PEG corona. (**Bc**) Schematic illustration of PEG-sheddable nanodrug targeting M2-like TAMs for tumor immunotherapy [102]. The pH-sensitive nanodrug with M2-targeting peptide (M2pep) was coated with a sheddable PEG corona. It was stable at pH 7.4 but cleavable in the acidic tumor microenvironment (TME) for active M2 targeting. A STAT6 inhibitor, AS1517499 (AS), and IKKβ siRNA were exposed for M2-to-M1 transpolarization for cancer immunotherapy. (**C**). Confocal micrographs of cellular backpacks attached to the surface of leukocytes (nucleus, blue; membrane, green; backpack, red) [103]. (**D**). Schematic illustration of neutrophil-mediated delivery of NPs to inflammatory tumor tissues induced by photosensitization (PS) [104]. Firstly, anti-CD11b antibody-coated NPs (NPs-CD11b) were constructed via biotin–neutravidin binding. Then, neutrophils were activated after tumor PS treatment and the intravenously injected NPs-CD11b were internalized by active neutrophils. Finally, NP-laden neutrophils infiltrated the tumor for drug delivery. (Image (**B**) is reprinted with permission from [102] (Copyright © 2020 American Chemical Society). Image (**D**) is reproduced with permission from [104] (Copyright © 2017 John Wiley & Sons, Inc.).

Apart from the above-mentioned methods that are used to encapsulate drugs into RBCs, the large surface area-to-volume ratio of RBCs is also beneficial for surface attachment of cargos via chemical conjugation or non-covalent binding mechanisms, such as electrostatic interactions, hydrogen bonding, van der Waals and/or hydrophobic forces [99]. As a paradigm of surface attachment for cargo loading, the idea of RBC hitchhiking (RH) is to adsorb therapeutic NPs onto RBC membranes for subsequent administration by intravenous (IV) or intra-arterial (IA) injection. This is a method that exhibits the synergistic effects of the “nano–micro–macro” combination (nanoscale drug carriers + microscale RBCs + macroscale IV/IA catheters) [100]. After entering the circulation, the NPs on RBCs are entrapped by pulmonary capillary endothelial cells when RBCs squeeze through the lung capillaries, leading to the passive dissociation of therapeutic agents from the surfaces of RBCs (Figure 3A). Therefore, this strategy is usually used to target the lungs and vessels rather than the spleen and liver [93,100]. Researchers have also attempted to employ RH for cerebrovascular targeting, producing a brain accumulation of 11.5% of the total injected dose of therapeutic agents [100]. Zelepukin et al. demonstrated that RBCs carrying certain small 100 nm-sized particles can be more effective to boost the delivery of non-targeted particles to the lungs, providing boosts up to a record high value of 120 times the delivery capacities of RBCs carrying sub-micron particles [101]. This result suggests that larger NPs are easier to be separated and removed from the surface of RBCs than smaller ones. Furthermore, the authors also screened RH with sub-200 nm-sized NPs and found that they were effective in inhibiting lung metastases of melanoma B16-F1 cells [101]. Therefore, they proposed the hypothesis that changing the affinity of RH complex formation may allow the targeting of different organs or at least the discrimination of different tissues with or without pathological changes [101].

Taken together, RBC-based DDSs take advantage of carrier RBCs to improve the circulating time, drug-loading capacity, and biocompatibility of therapeutic agents. However, as has already been mentioned, there are two major problems that remain unresolved after several years of preclinical research. On the one hand, the encapsulation procedure impairs the membrane integrity and function of RBCs to varying extents, reducing their biocompatibility and probably having systemic and local side effects in the body. On the other hand, bioactive drugs attached to the surface of RBCs could be desorbed by the shear force when drug-carrying RBCs pass the capillaries, which limits drug accumulation in target organs and thereby restricts therapeutic outcomes. However, this restriction could also be an unexpected advantage for the delivery of NPs with specific purposes, such as targeting blood- and endothelium-related diseases. Research on new ways to develop more efficient RBC-based DDSs with better safety profiles and therapeutic efficacies for the treatment of cancers is highly desirable.

#### 3.2.2. Macrophages and Monocytes

Macrophages are important innate immune cells that show inherent capabilities to detect, engulf, and digest invading pathogens or remove dying or dead cells and cellular debris, mainly based on the recognition of specific receptors termed pattern recognition receptors (PRRs). The ability of macrophages to sense chemotactic cues and home in on tumors is called chemotaxis and has been elucidated and exploited for targeted drug delivery to treat tumors [86]. Apart from intrinsic tumor tropism capacities, their superior phagocytic abilities also make macrophages the ideal natural drug carriers. Therapeutic NPs can be loaded into macrophages ex vivo by phagocytosis so that drug-bearing macrophages can then be re-injected into the body as “Trojan horses”, providing a potent platform for enhanced therapeutic efficacy in treating tumors [105]. Considering the possible biosafety risk of ex vivo preparation of drug-loading macrophages, researchers have developed an alternative in vivo internalization strategy for encapsulating drugs dependent on receptor-mediated phagocytosis for therapeutic intervention [106]. Xiao et al. developed a type of micellar nanodrug with M2 macrophage-targeted peptides hidden in the pH-sheddable PEG corona so that active targeting of M2 macrophages is triggered only in the acidic TME [102] (Figure 3B). Smart nanodrugs direct the transpolarization of M2 towards M1 macrophages via co-delivery of IKKβ siRNA and STAT6 inhibitors to suppress tumor growth and metastasis [102].

The entrapped therapeutic agents in phagosomes, especially some genetic drugs, may be degraded within destructive lysosomes; therefore, more research needs to be carried out to find a way to preserve drug stability and biological activity in the circulation and in target sites. Shields IV et al. constructed discoidal particles (referred to as “backpacks”) which can adhere to macrophage surfaces, thus evading phagocytosis for prolonged durations, and maintain the M1 polarization of tumor-associated macrophages (TAMs) to potentiate a robust anti-tumor response [103]. However, the cellular functions of macrophages may be compromised by the high surface densities of particles since the sufficient exposure of proteins on plasma membranes to microenvironmental stimuli is essential for certain cellular functions, such as ligand recognition and chemotaxis (Figure 3C).

Tumor-infiltrating macrophages possess intrinsic phenotypic plasticity allowing adaptation to specific TMEs and impacts on tumor progression. In turn, the TME can dictate macrophage polarization towards a favorable M2 phenotype with immunosuppressive and pro-tumorigenic properties from M0 and M1 phenotypes [107]. Compared to M2 macrophages, M1 macrophages are considered to support anti-tumor effects and have stronger phagocytic capabilities that would help to improve drug-loading efficacy. To maintain M1 polarization in the TME, IFN-γ-loaded “backpacks” can be bound to the surfaces of macrophages with sustained release of IFN-γ to treat murine mammary carcinoma [103]. Thus, M1 transpolarization from M2 TAMs in the TME emerges as a promising strategy for anti-tumor immunotherapy, and macrophage-based DDSs engineered with nanotechnology will provide a more effective cellular function-driven strategy for targeted anti-cancer therapy.

Monocytes are mononuclear phagocytes circulating in the bloodstream which can be recruited at every step during tumor progression. Classical monocytes are believed to be a major source of TAMs because they can be recruited to deep hypoxic areas of tumors [108]. Such a property confers on monocytes the desirable potential to deliver drugs into inaccessible areas inside tumors such as hypoxic and necrotic regions. Smith et al. indicated that, of all myeloid cells, only Ly-6C^hi^ monocytes displayed substantial single-walled carbon nanotube (SWNT) uptake within 2 h post-intravenous injection of SWNTs [109]. In contrast, other circulating white blood cells only took up negligible amounts of SWNTs [109]. This high selectivity of SWNT uptake by the Ly-6C^hi^ subset instead of the EPR effect is probably much more beneficial for treating solid tumors. Similarly, Yang et al. reported that COSA micelles, composed of chitosan and stearic acid, were selectively taken up by circulating Ly-6C^hi^ monocytes in a receptor-mediated way after intravenous administration [110]. It is difficult for injected particles to reach deep hypoxic regions given the lack of vessels in solid tumors; targeting Ly-6C^hi^ monocytes in the blood for drug delivery may overcome this limitation. In addition, flat, disk-like particles attached to the surface of monocytes can also avoid phagocytosis by monocytes, which can limit the toxic effect of NPs on monocytes [111].

#### 3.2.3. Neutrophils

Neutrophils, one of the primary kinds of effector cells in acute inflammation, lead the first wave of host defense against infection or tissue damage [112]. In humans, they are the most abundant (50–75%) type of leukocytes circulating in the bloodstream and require constant replenishment because of their short half-life [113,114,115]. Activated neutrophils hold great potential in cancer targeted drug delivery, having advantages similar to those of other immune cells, such as niche-targeting trafficking properties via intrinsic cell adhesion molecules on membranes [116]. Neutrophils also have distinct capabilities to travel to the brain and penetrate inflamed brain tumors which other cells cannot easily access [117]. For example, Xue et al. reported that paclitaxel (PTX)-loaded neutrophils could suppress postoperative glioma recurrence [118]. Firstly, PTX-loaded cationic liposomes were incubated with neutrophils in vitro to obtain PTX-loaded neutrophils by neutrophil internalization. Secondly, inflammatory factors released after surgical tumor resection guided the transmigration of the PTX-loaded neutrophils into the inflamed area of the brain. Finally, PTX was released from PTX-loaded neutrophils to induce cytotoxicity toward the recurring tumor cells to inhibit their proliferation and tumor relapse. This is an ideal design that makes full use of the physiological properties of native neutrophils to enhance the therapeutic potential of anti-cancer drugs. Nevertheless, NPs internalized into neutrophils ex vivo may also have some common flaws, such as reduced cell viability, insufficient cell numbers, and a risk of in vitro contamination. These concerns should be addressed to ensure the biosafety and efficacy of neutrophil-based DDSs.

Moreover, better therapeutic results may be achieved if activated neutrophils that can be used as drug delivery carriers can be produced incidentally during certain combination therapies. In preclinical trials, several strategies have been used in combination with neutrophils to improve the therapeutic efficacy of neutrophil-based DDSs, such as photothermal therapy (PTT), which could naturally create an acute inflammatory environment for the recruitment of neutrophils into tumor tissues [119,120]. Based on this phenomenon, Li et al. proposed a more advantageous strategy for in situ hitchhiking of circulating neutrophils for cancer treatment [121]. The researchers cloaked NPs with bacteria-secreted outer membrane vesicles to construct neutrophil-based delivery vehicles carrying nano-pathogenoids which could eliminate residual microtumors after PTT [121]. By this means, the number of neutrophils in tumors could be increased by up to 300–600% via PTT pretreatment, which dramatically improved therapeutic outcomes. Consequently, a single treatment produced a 60% tumor-free rate and a 97% tumor growth inhibition rate, and tumors were completely eradicated by repeated treatment [121].

The in situ hitchhiking strategy is practically advantageous. Once engineered nanomedicines can specifically target activated neutrophils in situ, therapeutic agents sequentially hijack neutrophils for targeted drug delivery. Nanomedicines with high binding affinity to neutrophils are critical for this active targeting process. In preclinical studies, numerous membrane proteins of neutrophils are potential candidates for this active targeting of NPs. For example, anti-CD11b antibody-coated NPs can facilitate engulfment by neutrophils in vivo because CD11b is highly expressed on activated neutrophils [122]. This neutrophil-based drug delivery enables therapeutic agents to reach TMEs along with neutrophil infiltration induced by photosensitization [104] (Figure 3D). In fact, the selective targeting of neutrophils via high-affinity ligands is still a challenge, owing to a close lineage similarity between neutrophils and other types of myeloid cells, such as monocytes, macrophages, and osteoclasts [123]. However, as the first responders, neutrophils initiate host defenses against various pathogens and could definitely serve as promising natural carriers to transport payloads from blood circulation to diseased tissues across the blood vessel barrier via chemotactic recruitment [114].

## 4. Pathogen-Based NPs

Pathogen-based DDSs can deliver a variety of drugs to target sites, taking advantage of inherent biomimetic properties and the immunogenicity of carriers [124,125,126,127]. For example, most cells, particularly phagocytes, can ingest pathogen particles by endocytosis and transport payloads to target sites [121,128]. There are many types of pathogens that can be engineered as agent carriers and numerous efforts have been made to use these pathogens for therapeutic delivery functions [129].

### 4.1. Viruses

Viruses are composed of genomic DNA or RNA and a protein coat capsid. They can package and transfer their nucleic acids into host cells for self-replication and exhibit intrinsic abilities to evade host immune surveillance by several means [130,131,132]. Viral vectors, such as adenoviruses, adeno-associated viruses, retroviruses, and lentiviruses, are regarded as superior natural nucleic acid vehicles because they can protect and carry their cargos for gene therapy, but their application remains a matter of debate due to biosafety concerns caused by their immunogenicity and off-target effects [133]. The genetic reprogramming of viruses, which replaces their genetic materials with therapeutic nucleic acids, has shown great potential to improve their biosafety and therapeutic efficacy [134]. Moreover, surface modifications to both the exterior and interior capsid surfaces, another crucial engineering approach, can also broaden the application of virus-based DDSs by improving pharmacokinetics and targeted delivery efficacy, while attenuating immune responses [133,135]. Numerous therapeutic agents can be effectively delivered to treat breast, melanoma, ovarian, and prostate cancers via these approaches [136]. In recent years, plant virus-based nanotechnologies have also been recognized for their possible applications in treating human diseases [137,138]. Plant viruses and certain bacteriophages may provide higher biosecurity, as the nucleic acids of these viruses cannot be integrated into the genomes of mammalian cells, avoiding possible infection, contaminations, and genomic mutations [135].

Virus-like particles (VLPs) are self-assembled particles with capsids that can be utilized as delivery carriers for various cargos, including small synthetic molecules, antigens, adjuvants, nucleic acids, peptides, and proteins [126,129]. Compared to conventional viral vectors, VLPs lack a viral genome and are considered to be safer carriers because they can only mimic the bioactivity of their parental virus and have comparable immunogenicity to the parental virus [129]. Different strategies, such as self-assembly processes, genetic engineering, infusion, and bioconjugation, have been developed to carry the payloads either inside the VLPs or on the outside membranes of capsids [139,140,141,142,143]. Taking advantage of the immunogenicity of viruses, VLPs have been frequently designed for vaccination or immunotherapy in cancer treatment [144,145]. In addition, VLPs also share the intrinsic tropisms of their parental viruses towards certain organs or tissues [146]. This characteristic of VLPs facilitates the development of VLP-based DDSs dependent on their natural targeting capabilities without requiring further engineering processes; for example, hepatitis B VLPs can be used to target the liver, JC VLPs to target glial cells, and papilloma and polyoma VLPs to target the spleen [147,148,149,150,151,152].

Virosomes, another category of virus-derived particles, are liposome-like NPs—phospholipid-bilayered vesicles with virus-derived surface glycoproteins and removed nucleocapsids [129,153,154]. Two unique influenza envelope proteins, haemagglutinin and neuraminidase, are important for virosome reconstitution and endow virosomes with excellent adjuvant properties for the production of various vaccines [154,155]. Virosomes can be modified by different types of antigen epitopes and can target different kinds of host cells [156]. Virosome-based vaccine delivery systems have been successfully developed against hepatitis A viruses and influenza viruses, but in vivo applications are limited due to their high immunogenicity [129].

**Figure 4:**
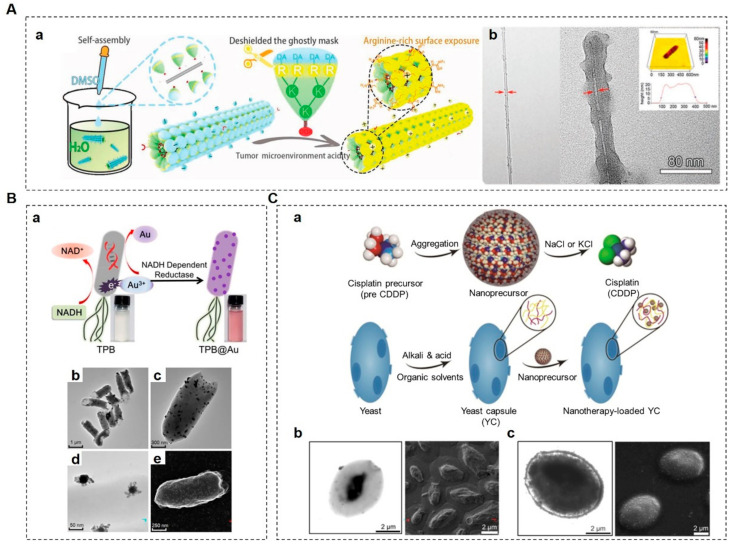
Pathogen-based drug delivery systems. (**A**). Construction of an artificial tobacco mosaic virus (ATMV). (**Aa**) Schematic illustration of supramolecular assembly fabrication of ATMVs. (**Ab**) TEM images of a single-walled carbon nanotube (SWNT) which was conjugated with an RGD peptide (SWNT-R) (left) and ATMVs (right). Atomic force microscopy (AFM) image of ATMVs (upper right) [159]. To build the ATMVs, SWNT-R scaffolds and capsid subunit mimetic dendrons (CSMDs) were co-dissolved in dimethyl sulfoxide (DMSO) and then the mixture was dropped into ultrapurified water under ultrasonic conditions to form tightly ordered arrays that closely mimicked the structure of tobacco mosaic virus. (**B**). Bacteria-based anti-tumor vehicles. (**Ba**) Biosynthesis mechanism of TPB@Au. AuNPs were adsorbed onto the thermally sensitive programmable bacteria (TPB) through enzymatic reduction to obtain TPB@Au. (**Bb**,**Bc**) TEM images of TPB@Au. (**Bd**) TEM image of AuNPs on the surface of TPB@Au. (**Be**) SEM image of TPB@Au [125]. (**C**) Biomimetic yeast microcapsule for anti-tumor therapy. (**Ca**) Schematic illustration of a nanoprecursor packaged into a yeast capsule (YC). A water soluble cis-diamminedichloro-platinum (CDDP) precursor (PreCDDP) was loaded into the interior of a YC and was simultaneously adsorbed on the YC wall largely by electrostatic forces. (**Cb**) TEM (left) and SEM (right) images of YCs prepared under optimized core-removing conditions. The core contents of YCs were partially removed, resulting in a collapsed structure. (**Cc**) TEM (left) and SEM (right) images of PreCDDP-loaded YCs. The interiors of YCs were largely filled with PreCDDP post-drug-loading and exhibited a plump morphology [160]. (Image (**A**) is reproduced with permission from [159] (Copyright © 2020 John Wiley & Sons, Inc.). Image (**B**) is reprinted with permission from [125] (Copyright © 2018 American Chemical Society).)

Over the past decades, researchers have been working to apply the merits of viruses to synthetic materials, while ensuring the biosafety of viral carriers for their successful use in drug delivery. Mimicry of the structural advantages and surface characteristics of viruses has been used to improve targeting efficiency and cellular uptake and to achieve mucus penetration, which is beneficial for drug delivery [157,158]. For instance, Wu et al. developed an artificial tobacco mosaic virus (ATMV) therapeutic agent to the mimic rod-shaped structures and infection process of tobacco mosaic virus (TMV) [159]. Negatively charged ATMVs with high-aspect ratio morphologies showed significant advantages, namely, long-term circulation, powerful tumor tropism, and robust oncolytic potency owing to their arginine–glycine–aspartate (RGD) modification and close structural resemblance to TMV [159]. They could not only destroy primary infected cells but also deeply infect solid tumor cells by cell-to-cell disseminations (Figure 4A).

Although these strategies have been extensively investigated in preclinical research, their clinical applications are often unsatisfactory. When virus-based drug delivery is used in vivo, the biosafety concern should be a top priority, and it is a matter that needs further investigation. Modification strategies to overcome this obstacle and reduce the off-target effects of virus-based DDSs are still in urgent demand.

### 4.2. Bacteria

The idea of using pathogenic bacteria as oncolytic agents to activate the immune system to fight against cancer has been raised for more than a century. However, infection-mediated treatment by oncolytic bacteria is usually accompanied by serious adverse effects, making this therapy controversial and limiting its use [161]. Recently, the use of bacteria as anticancer agents has been reproposed with the deep understanding of the TME and the development of bioengineering technology [162]. Therefore, bacterial strains with reduced bacterial virulence and attenuated immunogenicity but without compromised tumor targeting capability have been produced by genetic engineering for selective tumor treatment [162,163]. For instance, an auxotrophic strain was generated to compete with tumor cells for local nutrients and control tumor cell proliferation in vivo without causing serious systemic toxicity, while another strain with reduced bacterial virulence was generated by depletion of toxic genes with strongly reduced swarming but without attenuation of flagellar swimming and twitching motility [164,165]. In addition, recombinant bacteria can produce multiple therapeutic substances (e.g., cytotoxins, cytokines, tumor-associated antigens) for the treatment of cancer [125,166,167].

Genetically modified bacteria which carry therapeutic payloads, including but not limited to nucleic acids, cytotoxic agents, and enzymes, can serve as efficient drug delivery vehicles to enter the hypoxic and necrotic regions of solid tumors due to their invasive properties [168]. The passive behavior of bacterial carriers could be affected by various environmental stimuli, while their active migration towards more favorable conditions is guided by bacterial taxes, such as chemotaxis, phototaxis, thermotaxis, pH taxis, and aerotaxis [169,170,171,172]. For example, facultative and obligate anaerobic bacteria display innate tumor-targeting abilities, which enable them to penetrate deeply into solid tumors following a high-to-low gradient of oxygen concentration [173,174,175].

The biohybrid micromotors design concept has also been developed as an additional paradigm in bacteria-based drug delivery based on the self-propulsive capabilities of bacteria. In a recent study, Alapan et al. present a multifunctional biohybrid microswimmer with the potential to be used soon in in vivo medical applications [176]. This biohybrid system is composed of bioengineered motile bacteria and RBCs loaded with the anticancer drug DOX and superparamagnetic iron oxide NPs (SPIONs). This therapeutic agent has autonomous propulsion towards target tissues and can also be actuated under an oscillating magnetic field, demonstrating that bacteria-based biohybrid systems represent a potent tool for selective tumor-targeted delivery [176]. However, there are several limitations for biohybrid delivery systems. For instance, the loading of substances may interfere with the motion of biohybrid microbots, resulting in off-target effects, and the surface conjugation of NPs may limit drug-loading efficiency as well.

Alternative approaches using bacteria-derived microvesicles, such as bacterial ghosts (BGs) or outer membrane vesicles (OMVs), have been considered much safer than intact bacteria-based delivery systems because of reduced immunogenicity and pathogenicity [177,178].

BGs are empty and non-living cell envelopes of Gram-negative bacteria produced by the controlled expression of lysis gene E [179]. BGs preserve the entire surface structures of native bacteria for activating the innate immune response, offering a tremendous platform which not only provides efficient adjuvants for vaccines but also shows therapeutic potential in combination with versatile carriers for co-delivery systems [179,180]. For example, when combined with immunotherapies, BGs show excellent performance in priming dendritic cells (DCs) and releasing tumor antigen payloads, resulting in more robust activation of CD8^+^ T cells than can be achieved using lipopolysaccharide-based vectors [181].

OMVs are spherical buds on the outer membranes of Gram-negative bacteria filled with periplasmic content with sizes ranging from 20 to 400 nm in diameter [182,183]. OMVs are usually utilized as non-living complexes for vaccinations and drug delivery vehicles because of their immunomodulatory activities [183,184]. In order to investigate the potential of bacterial OMVs as therapeutic agents for cancer immunotherapy, Kim et al. demonstrated that systematically administered bacterial OMVs could fully eradicate established tumors by inducing long-term anti-tumor immune responses without obvious adverse effects [124]. Fan et al. developed an excellent design that took full advantage of bacterial OMVs for tumor treatments [125] (Figure 4B). Non-invasive, thermally sensitive programmable bacteria (TPB) were transformed with plasmids to express therapeutic protein tumor necrosis factor alpha (TNF-α) and then decorated with gold NPs (AuNPs) to obtain TPB@Au. After oral administration, the therapeutic agents could be protected by TPB in the gut and transported into tumor sites due to an anaerobic homing feature [125]. Inspired by the intrinsic adjuvant properties of parental bacteria, OMV-coated NPs have been employed to enhance immune responses [185]. Wang et al. constructed a bacterial vesicle–cancer cell hybrid membrane-coated NP which integrated hollow polydopamine (HPDA) NPs with OMV and B16-F10 cancer cell membranes [186]. The dual-functional NPs were used to improve anti-tumor efficacy toward melanoma by exploiting the synergistic effects of OMV-mediated immunotherapy and HPDA-mediated photothermal therapy [186].

It is well known that an ideal drug-delivery system for cancer therapy should deliver the substance selectively to tumor sites in order to maximize tumor-killing effects without harming healthy tissues. Genetically engineered bacteria can help to achieve this goal. Taking advantages of their active propulsion and environmental sensing capabilities, we can steer bacteria to specific regions inside the body, which proves that bacteria can serve as good tumor-targeting and drug-loading vehicles.

### 4.3. Fungi

*Saccharomyces cerevisiae* is non-hazardous and has been extensively used in the food and beverage industry [187]. Due to their good safety and particular cell wall components, these fungi are also some of the most studied for DDS construction. Yeast cells whose membranes consist of β-1,3-D-glucan polymers associated with mannose-containing proteins and chitin can be recognized by dectin-1, a membrane receptor which is expressed on several types of antigen-presenting cells (APCs) (e.g., macrophages and dendritic cells) [188,189]. Some researchers have taken advantage of this feature of yeast cells to develop a promising strategy for targeting atherosclerotic plaques or tumors via an oral route [160,190]. After oral administration, yeast cells are taken by microfold cells through Peyer’s patch and are transported via the lymphatic route to the systemic circulation for efficient drug delivery [191]. The advantage of this strategy is that the orally administered particulate vehicles transported through the intestinal lymphatic system can bypass the hepatic first-pass metabolism, ensuring higher concentrations of therapeutic agents in the circulation and target tissues [192].

Yeast microcapsules (YCs) can be prepared from yeast cells by treatment with alkalis, acids, and organic solvents, resulting in minimal cytoplasmic contents and the preserved cellular morphologies of the yeasts [189]. YCs are porous and hollow microspheres which can serve as vehicles for various cargos, such as genes, proteins, and drugs, that can be efficiently encapsulated by electrostatic interactions [190]. Zhou et al. demonstrated that orally delivered drug-laden YCs accumulated in human A549 lung carcinoma xenografts in mice and showed desirable anti-tumor effects (Figure 4C). Therefore, YC-related biomimetic approaches can probably serve as an effective strategy for targeted delivery of chemotherapies by oral administration [160].

In addition, YCs can also be hydrolyzed into small fragments after internalization by macrophages due to their biocompatibility and biodegradability [193]. Furthermore, YCs are non-pathogenic, even though they can induce immunological responses in mammals owing to their β-glucan constituents, known as immunomodulatory compounds, which possess strong adjuvant properties [188,193]. Moreover, YCs exhibit good safety profiles after long-term oral administration, which is extremely important for the management of chronic diseases [190]. All of the above outstanding characteristics make YCs a promising drug delivery platform for tumor-targeted treatment.

## 5. Biohybrid Micro-/Nanomotors

Inspired by fascinating biomolecular motors and movable organisms, scientists have developed self-propelled micro- and nanomotors (MNMs) which can effectively convert surrounding chemicals or external energies into driving forces for autonomous motion [194]. Compared to ordinary NPs, these MNMs are considered to have great potential for tumor-targeted delivery and tissue penetration by overcoming biological barriers in an autonomous manner driven by propelling forces [27]. Generally, there are two main categories of MNMs. The first is that of chemically propelled MNMs, which utilize local chemicals to generate driving forces, such as bubble propulsion, self-diffusion, and electrophoresis, via specific catalytic or spontaneous reactions in the surrounding environment [195,196,197]. The other is that of fuel-free MNMs, which can be propelled by external fields, such as magnetic, electric, ultrasonic, and optical fields, showing alternative ways in which autonomous motion can be induced in MNMs [194].

**Figure 5:**
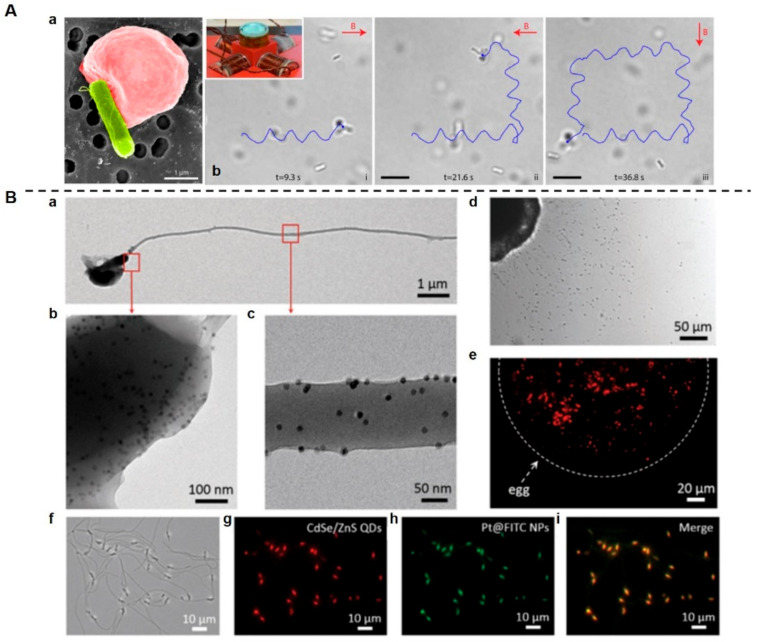
Biohybrid micro-/nanomotors. (**A**). RBC microswimmers for active cargo delivery. (**Aa**) SEM image of an RBC microswimmer with an attached bacterium (pseudo-colored red, RBC; pseudo-colored green, bacterium). RBC microswimmers were constructed through the non-covalent interaction of biotin-functionalized RBCs with streptavidin-coated motile bacteria. (**Ab**) The RBC microswimmer changed swimming direction when the magnetic field direction was changed (i–iii) [176]. Red arrows indicate the direction of the magnetic field. The inset shows the setup used for magnetic steering of the RBC microswimmers. Scale bars = 10 μm. (**B**). Free-swimming-functionalized sperm micromotors (FSFSMs) for efficient drug-loading and self-propulsion. (**Ba**–**c**) TEM images of FSFSMs loaded with iron oxide (Fe_2_O_3_) NPs. (**Bd**) After incubating with the FSFSMs for 10 min, egg cells were surrounded by swarming FSFSMs. The functionalized sperm cells maintained their chemotactic ability to sense egg cells. (**Be**) Fluorescence image of an accumulation of FSFSMs on the egg surface. (**Bf**) Microscopic brightfield and (**Bg**–**i**) fluorescence images of the same sperm motor group functionalized with multiple payloads: CdSe/ZnS QDs (**Bg**), Pt@FITC NPs (**Bh**), and (**Bi**) merged by two fluorescence channels [199]. (Image (**A**) is reproduced with permission from [176] (Copyright © 2018, The American Association for the Advancement of Science). Image (**B**) is reproduced with permission from [199] (Copyright © 2017 John Wiley & Sons, Inc.).)

In recent years, much attention has been paid to artificial MNMs, the behaviors of natural living systems inspiring improvements in tumor-targeting efficiency. Apart from the aforementioned bacteria-based MNMs, sperm-based MNMs are also important in this field of study, due to their good biocompatibility and autonomous motility [194]. Sperm, the specialized male reproductive cells, possess chemotactic properties and excellent self-propulsive capabilities, generated by the beating of the sperm flagella [198]. Biohybrid MNMs constructed by integrating sperms into artificial materials may serve as useful exploratory tools for targeted drug delivery. As an example of the application of free-swimming functionalized sperm micromotors (FSFSMs), Chen et al. took advantage of the endocytosis ability and chemotactic swimming behavior of sperm cells to develop an intelligent and self-guided biomotor, loading multiple synthetic payloads with different characteristics into natural sperm cells [199] (Figure 5).

However, micromotor navigation in complex blood vessels may be disturbed by many factors, such as blood velocity, fluid viscosity, and blood content [194]. To overcome these hurdles, Xu et al. developed streamline-horned cap (SHC) hybrid sperm micromotors which could efficiently control the directed swim against flowing blood and cargo delivery [200]. The high propulsive force of the hybrid sperm micromotors was generated by a combination of rheotaxis and thigmotaxis inherited from the sperm and the magnetic guidance from the coupled horned caps which were loaded with heparin [200]. Although the hybrid sperm micromotors showed promise for cargo delivery against blood flow, the propulsive force was not yet high enough to overcome the blood flow in large arteries [200]. In addition, to avoid the motility impairment of sperms induced by antisperm antibodies (AsAs) in body fluids, Chen et al. reported another kind of biohybrid sperm microrobot [201]. They encapsulated sperm cells within metal organic frameworks (MOFs) and zeolitic imidazolate framework-8 (ZIF-8) NPs (ZIFSpermbot) to fulfil active drug delivery and avoid the biological threats from AsAs [201].

Biohybrid MNMs with inherent properties, including remarkable speed, large cargo-towing ability, and precise motion control, are powerful candidates for improving cancer-targeted treatment [176,200]. Within certain biological environments, these MNMs can sustain propulsive capabilities as long as the necessary fuels exist. However, in practice, the efficient propulsion of MNMs is often hindered by the absence of inertial forces, which is common for macroscale objects [202]. Therefore, biohybrid MNMs should be designed to collect as much chemical fuel from surrounding environments as possible in order to overcome the limitation mentioned above. In addition, available external forces are also necessary and powerful supplements for ensuring the successful autonomous motion of biohybrid MNMs.

## 6. Conclusions

Biohybrid therapeutic agents preserve features and functions from parental cells to overcome various biological barriers for the improvement of diagnosis and treatment of cancers. All the functionalized DDSs with intrinsic properties summarized above can serve as potent platforms in prolonging circulation time, increasing specific targeting capability, and enhancing the extensive immunomodulatory activities of various drugs.

In preclinical studies, numerous proofs of concept are continuously emerging, while there still remains a relatively long way to go before the clinical translation of these ideas can be achieved. This predicament is largely due to the complexity and heterogeneity of tumors and incomplete understanding of the relevant nano-biointeractions. Therefore, bioinspired and biomimetic nanomedicines are still in their infancy and there are several challenges that need to be overcome to obtain the desired efficacies in anti-cancer treatment.

First, overall, biohybrid formulations remain to be investigated and standardized. The pharmacokinetic and pharmacodynamic characteristics of synthetic materials are as important as the biofunctions of natural materials and should be further profiled and defined. Second, mimicking key biofeatures of natural materials to preserve specific biological morphologies, structures, and functions is also required to construct efficient biomimetic nanomedicines. However, we should clearly note that the precise architecture of creatures is too complicated for replication by only physical and chemical methods. Therefore, we have to seek other alternative methods to maximize the efficacy of biomimetic nanomedicines, considering the inevitable compromises of functionality that are associated with these approaches. Third, unlike well-defined synthetic materials, the reproducibility, biosafety, and scale-up manufacturing of biomimetic nanomedicines must be fully considered before translation to clinical practice.

The field of biomimetic nanomedicines is developing rapidly. Future advances in this field will rely on deep understanding of nano-biointeractions, delivery mechanisms, and advanced synthesis techniques. Meanwhile, controllability and large-scale production capabilities should also be improved before clinical application.

## Figures and Tables

**Table 1:** Summary of cell membrane-camouflaged nanomedicines for cancer therapy.

Cell Source	Synthetic Carrier	Therapeutic Agent	Fabrication Methods	Cancer Model	Unique Advantages	Refs.
RBC	Fe_3_O_4_ MNs	-	Extrusion	CTCs	Reduced non-specific protein adsorption; Prolonged circulation time	[74]
	BSA PAAV-SNO	10-HCPT; ICG IR1061 1-MT	Sonication and extrusion	HeLa 4T1	Synergistic chemo-PTT; Immune evasion ability	[75,76,77]
Macrophage	Polymer	PTX	Sonication and extrusion	MDA-MB-231	Tumor-homing ability; Controlled release	[78]
TAAM	UCNP	RB	Extrusion	4T1	TME targeting	[23]
Neutrophils	PLGA	PTX	Sonication and extrusion	SKOV3	Prolonged circulation time; Enhanced accumulation	[79]
Platelet	Fe_3_O_4_	SAS	Extrusion	4T1	Effective ferroptosis; Mild immunogenicity	[80]
NK cell	PLGA	TCPP	Sonication and extrusion	4T1	M1-Mφ polarization; Activated effector T cells	[81]
T cell	HA-SS-VES	Curcumin	Extrusion	B16	Bind to PD-L1; Membrane escape effect	[67]
Tumor cell						
CT26	Bi NPs	-	Extrusion	CT26	Long-term circulation	[82]
B16	USIO NPs	DOX	Extrusion	B16	Homotypic targeting; Immune escape	[13]
bEnd.3	(PTX)NS	PTX	Extrusion	bEnd.3	BBB penetration	[30]
Hybrid membrane						
RAW264.7 4T1	PLGA	Met; siFGL1; DOX	Sonication	4T1	Lysosomal escape; Targeting metastasis	[68,72]
B16F10; 4T1; M1-Mφ; platelets	-	-	Sonication and extrusion	B16F10; 4T1	Increased affinity to CD47; M2-to-M1 repolarization	[83]
RBC; MCF-7	Melanin NPs	-	Sonication and extrusion	MCF-7	Prolonged circulation time;Homotypic targeting	[84]

Abbreviations: RBC, red blood cell; MNs, magnetic nanoparticles; CTCs, circulating tumor cells; BSA, bovine serum albumin; PAAV-SNO, S-nitrosothiols (SNO)-pendant copolymer (poly(acrylamide-co-acrylonitrile-co-vinylimidazole)-SNO copolymer; PTT, photothermal therapy; RES, reticular endothelial system; TAMM, tumor-associated macrophage membrane; UCNP, upconversion nanoparticle; TME, tumor microenvironment; RB, Rose Bengal; PLGA, poly (lactic-co-glycolic acid); SAS, sulfasalazine; PTX, paclitaxel; NK, natural killer; TCPP, 4,4′,4″,4‴-(porphine-5,10,15,20-tetrayl) tetrakis (benzoic acid); Mφ, macrophage; HA-SS-VES, hyaluronic acid-grated-disulfide bond-vitamin E succinate; PD-L1, programmed cell death ligand-1; CT26, mouse colon cancer CT26 cells; Bi, bismuth; NPs, nanoparticles; B16, mouse melanoma cell line; USIO, ultrasmall iron oxide; DOX, doxorubicin; NS, nanosuspensions; BBB, blood brain barrier; Met, metformin; siFGL1, small interfering fibrinogen-like protein 1.

## Data Availability

Not applicable.
